# Relationship between the Interannual Variations of Summer Convective Afternoon Rainfall Activity in Taiwan and SSTA(Niño3.4) during 1961–2012: Characteristics and Mechanisms

**DOI:** 10.1038/s41598-019-45901-w

**Published:** 2019-06-28

**Authors:** Wan-Ru Huang, Ya-Hui Chang, Po-Han Huang

**Affiliations:** Department of Earth Sciences, National Taiwan Normal University, Taipei, Taiwan

**Keywords:** Atmospheric science, Climate-change impacts

## Abstract

This study examines the interannual variation of the Convective Afternoon Rainfall (CAR) activity (including frequency and intensity) in Taiwan during the summers (JJA) of 1961–2012 with a focus on identifying its relationship with the changes in sea surface temperature anomalies over the Niño3.4 region [SSTA(Niño3.4)] and the underlying physical mechanisms. Our analyses show that during the colder (warmer) phase of SSTA(Niño3.4), the subtropical high system over the region east of Taiwan is enhanced (weakened), the local surface wind convergence is enhanced (weakened), and the local thermal instability is enhanced (weakened), which facilitates (suppresses) the formation of CAR in Taiwan. This consistent negative relationship between the interannual variation of CAR frequency in Taiwan and SSTA(Niño3.4) occurs throughout 1961–2012. In contrast, the relationship between the interannual variation of CAR intensity in Taiwan and SSTA(Niño3.4) changed from positively correlated to negatively correlated in the late 1980s. This change is attributed to the change in the moisture supply for maintaining the CAR intensity in Taiwan from an increase (decrease) in the warmer (colder) phase of SSTA(Niño3.4) before the mid-1980s to the opposite after the late 1980s. These findings highlight how the rainfall characteristics in East Asia may change in response to changes in SSTA(Niño3.4).

## Introduction

**Figure 1:**
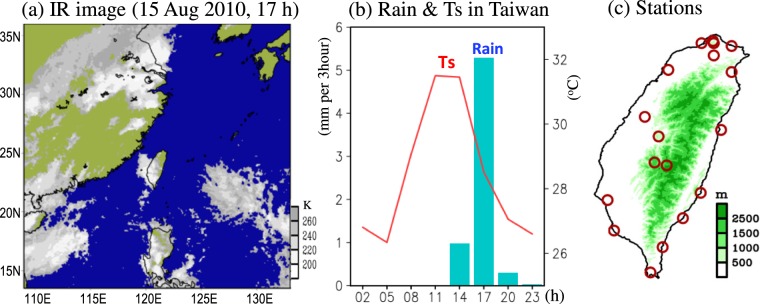
(**a**) GMS IR cloud image for an example of Convective Afternoon Rainfall (CAR) event that occurred on 15 August 2010, 17 h (local time) in Taiwan. The IR data is obtained from Gridded Satellite B1 (i.e. GridSat-B1) Observations, available at https://www.ncdc.noaa.gov/gridsat/. (**b**) Related time series of 3-hourly rainfall (bars) and surface temperature (Ts, red line) averaged from 18 standard stations in Taiwan for the event shown in (**a**). The red circles in (**c**) mark the locations of the 18 standard stations. The software used to create all maps in this figure is the Grid Analysis and Display System version 2.1.1.b0 (i.e. GRADS v2.1.1.b0), available at http://cola.gmu.edu/grads/downloads.php.

**Figure 2:**
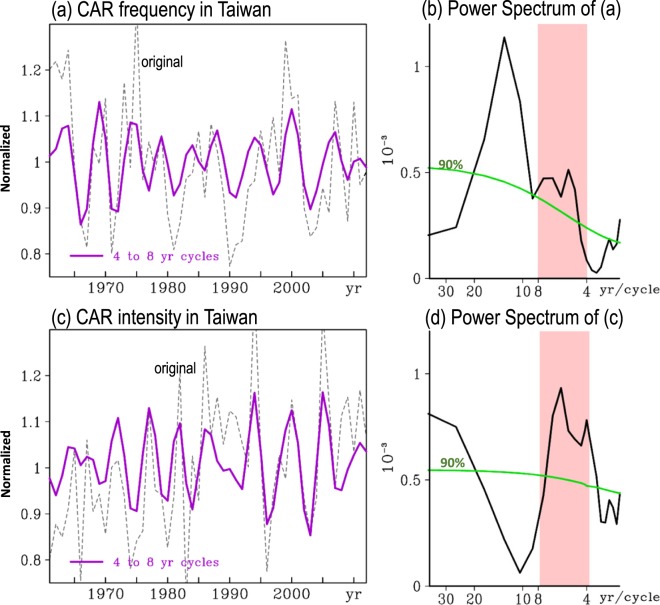
(**a**) Temporal variation of normalized CAR frequency (thin dashed line) averaged from all 18 standard stations in Taiwan and the related 4-to-8-year bandpass filtered cycles (purple line). (**b**) Power spectrum analysis of normalized CAR frequency in (**a**). (**c**,**d**) are similar to (**a**,**b**) but for the change in normalized CAR intensity. The normalization is performed by dividing the value of an analyzed variable by its climatological mean value during JJA of 1961–2012. In (**b**,**d**), the pink zones indicate the 4-to-8-year windows, and the values above the green lines are significant at the 90% confidence interval. This figure is modified from Fig. 2 of Huang *et al*.^5^.

In Taiwan, the Convective Afternoon Rainfall (CAR) event (Fig. 1a), which generally includes a diurnal rainfall maximum in the afternoon after the local thermal heating maximum (Fig. 1b), is the most common weather system during the summer (June, July and August; JJA)^1,2^. The temporal variations of CAR activity (including frequency and intensity) in Taiwan operate over multiple timescales^3–8^. Huang *et al*.^5^ showed that the CAR activity in Taiwan during JJA of 1961–2012 were characterized by variations at interdecadal (i.e., 10-to-20-year) and interannual (i.e., 4-to-8-year) timescales (Fig. 2; explained later). However, Huang *et al*.^5^ only explained the physical mechanisms for the long-term variations (>10 years) but not the interannual variations of CAR activity in Taiwan. It was demonstrated by Huang *et al*.^5^ that, with exception of the local dynamic and thermodynamic processes, the 10-to-20-year variations of CAR activities in Taiwan are affected by the changes of the remote sea surface temperature anomaly (SSTA) over the tropical Pacific region. Unlike Huang *et al*.^5^, who focus on the longer timescale variations, this study aims to determine whether SSTA over the tropical Pacific region has played a role in modulating the 4-to-8-year variations of CAR activity in Taiwan during JJA of 1961–2012.

The summer rainfall in Taiwan is greatly influenced by the variations of the East Asia summer monsoon (EASM) circulation^9,10^ and the related moisture transport^4,5,11,12^. Many studies have shown that the temporal variations of the EASM circulation are mainly characterized by quasi‐biennial and 3-to-7-year period oscillations and have suggested that the interannual variation of SSTA over the tropical Pacific regions is an important factor in modulating the EASM circulation^13–22^. Other studies^23,24^ have shown that there is a quasi-4-year coupling between the El Niño-Southern Oscillation (ENSO) and the variation of moisture transport over the EASM region. Recently, Li *et al*.^24^ further indicated that the interannual variation of moisture transport over the EASM region became more closely related to ENSO after the 1990s. In contrast, by examining the changes in the summer western North Pacific subtropical high (WNPSH) during 1948–2012, He and Zhou^25^ found that the interannual variability in the WNPSH has been more strongly regulated by the SSTA over the equatorial central Pacific and the maritime continent since the 1990s. Lee *et al*.^26^ also indicated that the changes of the SSTA in the tropical Pacific and the Indian Ocean impact the changes of the WNPSH. Because the changes in the EASM circulation, moisture transport and WNPSH are the most important factors that affect the CAR formation in Taiwan^10,27,28^, we then hypothesize based on these documented studies that (1) there might be a close relationship between the interannual variation of CAR activity in Taiwan and SSTA over the tropical Pacific regions, and (2) this relationship might have changed after the 1990s. These two hypotheses will be examined herein.

Recently, Chen *et al*.^6^ examined the relationship between the SSTA and the interannual variation of the summer afternoon thunderstorms in the Taipei basin (northern Taiwan) for 1993–2013. They noted that the summer afternoon thunderstorm activity in the Taipei basin is maintained by the warm, moist air transported by the southwesterly monsoon flow across Taiwan and that the monsoon southwesterlies are wetter (drier) during the cold (warm) SSTA over the Niño3.4 region [SSTA(Niño3.4)]. In contrast to Chen *et al*.^6^, who only focus on the Taipei basin after 1993, the main objective of this study is to investigate the possible relationship between SSTA(Niño3.4) and the CAR activity (including frequency and intensity) estimated for all of Taiwan over a longer time period (i.e., 1961–2012 JJAs) and to understand its underlying physical explanations. Examinations of these issues can lead to a better understanding of the characteristics and maintenance mechanisms of the interannual variation of CAR activity in Taiwan under long-term climate change.

## Data and Methods

### Data

Following Huang *et al*.^5^, the analyses utilize 3-hourly rainfall data extracted from 18 conventional meteorological stations (Fig. 1c; https://dbar.pccu.edu.tw/) over Taiwan during JJA of 1961–2012. The observed monthly SSTA are obtained from the National Oceanic and Atmospheric Administration (NOAA) Extended Reconstructed Sea Surface Temperature (ERSST) v3b dataset^29^ (https://www.esrl.noaa.gov/psd/data/gridded/data.noaa.ersst.v3.html), which has a spatial resolution of 2° longitude × 2° latitude. Additionally, meteorological data (e.g., wind fields, temperature, humidity) are extracted from the 55-year Japanese ReAnalysis (JRA-55)^30^ (https://rda.ucar.edu/datasets/ds628.0/). The 6-hourly JRA-55, which has a spatial resolution of 1.25° longitude × 1.25° latitude, has been demonstrated to represent most of the general characteristics of the atmospheric features associated with diurnal rainfall formation over East Asia^31^, including Taiwan^5^. For comparison with the 3-hourly surface observations, the 6-hourly JRA-55 data are linearly interpolated into 3-hourly data.

### Definition of CAR frequency and CAR intensity

The definitions of CAR frequency (i.e., the number of CAR days per JJA) and CAR intensity (i.e., the average rain rate during a CAR day) follow the procedure described in Huang *et al*.^5^. After the CAR frequency and CAR intensity have been calculated for all 18 stations, an equally weighted average is applied to the normalized CAR frequencies and CAR intensities from all 18 stations to obtain the mean CAR frequency and mean CAR intensity for all of Taiwan. The normalization is performed by dividing the value of an analyzed variable by its climatological mean value during JJA of 1961–2012. For additional details about identifying the CAR frequency and CAR intensity, please refer to Huang *et al*.^5^.

### Statistical methods

The vertically integrated specific humidity (denoted q_vint) is obtained based on Eq. (1):

1 $${\rm{q}}\_{\rm{vint}}=\frac{1}{g}({\int }_{300}^{Ps}\,q\,dp),$$

where g is gravity, p is the pressure, and q is the specific humidity. The moisture flux is calculated based on Eq. (2):

2 $${\rm{Q}}=\frac{1}{g}({\int }_{300}^{{p}_{s}}\,{\rm{Vq}}\,{\rm{dp}}),$$

where V denotes the horizontal wind vectors. Following Huang and Chang^7^, the potential function (χ_Q_) of the moisture flux is obtained by solving the Poisson equation in Eq. (3):

3 $${\nabla }^{2}{\chi }_{{\rm{Q}}}=\nabla \cdot {\rm{Q}}.$$

The extraction of the 4-to-8-year variability from an analyzed variable was performed using the Hamming-windowed bandpass method, which was developed to preserve the edges of filtered time series^32^. To help clarify the relationship between the examined variables, a 21-year running correlation is applied to the related examinations following the methods of Gershunov *et al*.^33^. The statistical significances of the analyzed variables are determined by a two-tailed Student’s *t*-test^34^ based on the effective degree of freedom. The method used to determine the effective degree of freedom follows Chen and Chen^35^. The criterion of significance is defined at the 90% confidence interval; this criterion has been adopted by many studies for identifying the existence of interannual variations in various variables^36^.

Throughout the study, the analyses are presented in Taiwan local time, which is universal time +8 h (i.e., 08 h corresponds to 00 UTC). The climatology is computed based on the variations over the time periods of JJA for 1961–2012. The composites of the active/inactive CAR activity and the warm/cold phases of SSTA(Niño3.4) are computed based on years with the related filtered time series passing the criteria of above/below the 0.5 standard deviation (STD).

## Results

### Relationship between CAR frequency and CAR intensity

Figure 2 shows the time series of the normalized CAR frequency (Fig. 2a; dashed line) and CAR intensity (Fig. 2c; dashed line) averaged for all of Taiwan during JJA of 1961–2012^5^. According to Huang *et al*.^5^, these time series consist of multiple timescale variations, including a clear increasing linear trend in CAR intensity (significant at the 90% confidence interval). Application of a power spectrum analysis shows that both the CAR frequency and CAR intensity have clear oscillating signals in the 4-to-8-year window (Fig. 2b,d)^5^. The temporal correlation coefficient between the two 4-to-8-year bandpass filtered (hereafter, filtered) time series in Fig. 2a,c during JJA of 1961–2012 is close to zero (~−0.04), suggesting that the filtered CAR frequency might not related to the CAR intensity. However, an examination of the 21-year running correlation between the filtered CAR frequency and CAR intensity shows that prior to the mid-1980s, the CAR frequency is negatively correlated with the CAR intensity, whereas the CAR frequency is positively correlated with CAR intensity after the late 1980s (Supplementary Fig. S1). This changing relationship, which has not been pointed out in Huang *et al*.^5^ and other studies, is not found if the temporal correlation is calculated for the entire period of 1961–2012. Moreover, this observation implies that either the filtered CAR frequency or the filtered CAR intensity in Taiwan has experienced a changing relationship with SSTA(Niño3.4) during JJA in 1961–2012. This inference is examined below.

### Relationship between CAR activity and SSTA(Niño3.4)

**Figure 3:**
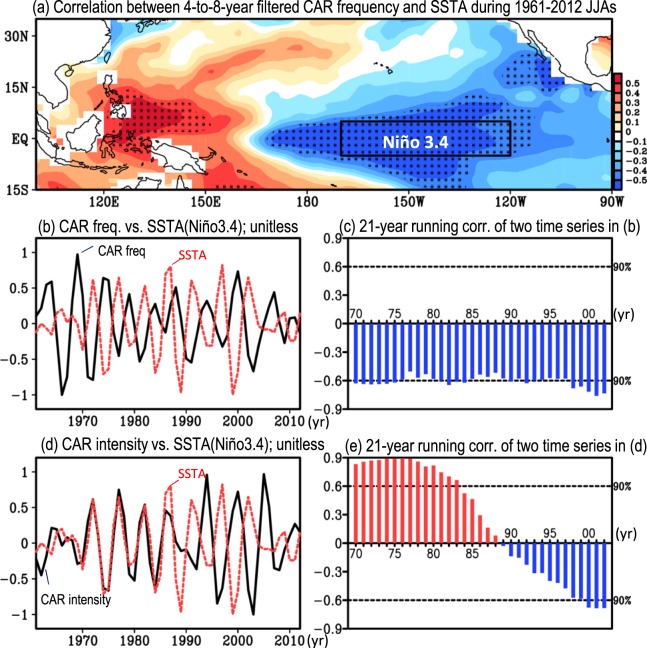
(**a**) Horizontal distribution of the correlations between the 4-to-8-year bandpass filtered CAR frequency in Taiwan and SSTA during JJA of 1961–2012. The areas with correlations that pass the 90% confidence interval are dotted. (**b**) Filtered time series of CAR frequency (black line) and SSTA(Niño3.4) [i.e., area-averaged over the boxed area in (**a**)] (red line). For comparison, both variables in (**b**) are unitless (i.e., divided by the maximum amplitude during JJA of 1961–2012). (**c**) The 21-year running correlation between the two filtered time-series in (**b**). On the x-axis, the year represents the mid-point of the 21-year window (e.g., 70 represents the running window of 1961–1981). (**d**,**e**) are similar to (**b**,**c**) but for the relationship between the filtered CAR intensity and SSTA(Niño3.4).

What is the relationship between the filtered CAR frequency in Taiwan and SSTA(Niño3.4)? Using the filtered CAR frequency in Taiwan to correlate the filtered SSTA during JJA of 1961–2012, Fig. 3a shows that there are significant negative correlation coefficients over the Niño3.4 region, suggesting that the CAR frequency in Taiwan tends to occur more (less) frequently during the colder (warmer) phase of SSTA(Niño3.4). This can be clarified by directly comparing the temporal variations of the filtered CAR frequency in Taiwan and SSTA(Niño3.4) (Fig. 3b). Furthermore, by examining the 21-year running correlation between the two filtered time series of Fig. 3b, we note that the observed negative relationship between the filtered CAR frequency in Taiwan and SSTA(Niño3.4) existed in JJA throughout all of 1961–2012 (Fig. 3c). In contrast, the relationship between the temporal variations of the filtered CAR intensity in Taiwan and SSTA(Niño3.4) (Fig. 3d) appears to have changed from positively correlated before the mid-1980s to negatively correlated after the late 1980s. To clarify the change in this relationship, the 21-year running correlation between the filtered CAR intensity in Taiwan and SSTA(Niño3.4) is computed; the results (Fig. 3e) confirm that the relationship between these two parameters changed in the late 1980s.

Notably, similar phase relationships between the interannual variations of CAR activity and SSTA(Niño3.4) can be found in the related examinations that either use (1) the time series obtained by simply removing the variations ≥10 years from the total time series (see Supplementary Fig. S2) or (2) the filtered time series of CAR activity extracted from each individual station (see Supplementary Fig. S3) to compare with the variations of SSTA(Niño3.4). These results support the hypothesis that the relationships between the interannual variations of CAR activity in Taiwan and SSTA(Niño3.4), seen in Fig. 3b–e, are robust.

On the other hand, it was known that the ENSO changed from a more Eastern-Pacific (EP) type of ENSO to a more Central-Pacific (CP) type of ENSO in the 1990s^37–42^, implying that the major center of the variation in SSTA patterns over the tropical Pacific regions changed in the 1990s. Thus, it is questioned that the changing relationship shown in Fig. 3d–e may not be real but rather a misunderstanding caused by the selection of the SSTA domain. To answer this question, we examine the horizontal distribution of the temporal correlation between the filtered CAR intensity in Taiwan and the SSTA over two selected time periods: pre-1985 (i.e., JJA in 1961–1985) and post-1990 (i.e., JJA in 1990–2012) (Supplementary Fig. S4). As shown in Fig. S4, for both time periods, the correlation values (despite the change in sign) that pass the significant test are all located over the Niño3.4 region and do not shift to the Central-Pacific region. Therefore, it is likely that the observed changing relationship between the filtered CAR intensity in Taiwan and SSTA(Niño3.4) is real rather than a misunderstanding caused by the selection of domain. Possible explanations for this change along with the consistent negative relationship between the filtered CAR frequency in Taiwan and SSTA(Niño3.4) are discussed below.

### Mechanism for the relationship between CAR frequency and SSTA(Niño3.4)

What causes the consistent negative relationship between the filtered CAR frequency in Taiwan and SSTA(Niño3.4) during JJA of 1961–2012? To answer this question, we examine the changes in the thermodynamic conditions that are important to the formation of CAR over Taiwan.

**Figure 4:**
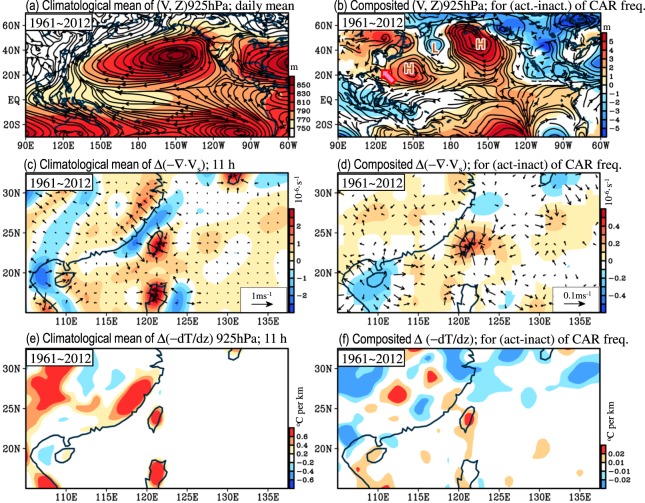
(**a**) Climatological mean of low-level circulation at 925 hPa [i.e., V(925 hPa)] and related geopotential height [i.e., Z(925 hPa); shaded] averaged during JJA of 1961–2012. (**b**) Differences in composited (V, Z) at 925 hPa between the active (>0.5 standard deviation; STD) and inactive (<0.5 STD) years of the filtered CAR frequency in Taiwan (i.e., black line in Fig. 3b) during JJA of 1961–2012. In (**b**), “H” and “L” denote the high (i.e., anticyclonic) and low (i.e., cyclonic) systems mentioned in the manuscript, respectively. The arrow in (**b**) indicates the southerly wind anomaly. (**c**,**d**) are similar to (**a**,**b**) but for the daytime surface wind convergence [i.e., Δ(−∇·V_s_); Δ denotes that the daily mean has been removed] at 11 h, Taiwan local time. (**e**,**f**) are similar to (**c**,**d**) but for the thermal instability [i.e., ${\rm{\Delta }}(\,-\,\frac{dT}{dz})$] at 925 hPa, 11 h. The calculations of Δ(−∇·V_s_) and ${\rm{\Delta }}(\,-\,\frac{dT}{dz})$ follow the method of Huang *et al*.^5^. In (**b**,**d**,**f**), only the areas with values that pass the 90% confidence interval are shown.

Climatologically, the low-level northern hemisphere summer circulation is characterized by a subtropical high over the western North Pacific (i.e., WNPSH), and Taiwan is modulated by the WNPSH (see Fig. 4a). A comparison of Fig. 4a,b shows that CAR in Taiwan tends to occur more frequently when the subtropical high over the area east of Taiwan (centered at 150°E, 25°N) is enhanced^6,43^. Previous studies^4,5,11^ have also shown that CAR generally occurs when Taiwan is located in the “west rim” of an intensified WNPSH, where the southerly wind component is generally enhanced^5^. Huang and Chen^4^ further noted that the enhanced southerly wind over the west of the intensified WNPSH tends to keep the synoptic frontal system (generated over the mid-latitude regions) in the area north of 25°N and leads to more weak-synoptic atmospheric conditions and more CAR events being observed around Taiwan. Moreover, when Taiwan is under weak-synoptic atmospheric conditions, the local daytime sea breeze circulation in response to the local thermal instability is generally more active than usual to support the formation of CAR^5,6^. Consistent with these studies^5,6^, the comparison between Fig. 4c,d (Fig. 4e,f) shows that CAR in Taiwan tends to occur more frequently under atmospheric conditions with enhanced local daytime sea breeze convergence (enhanced local thermal instability).

**Figure 5:**
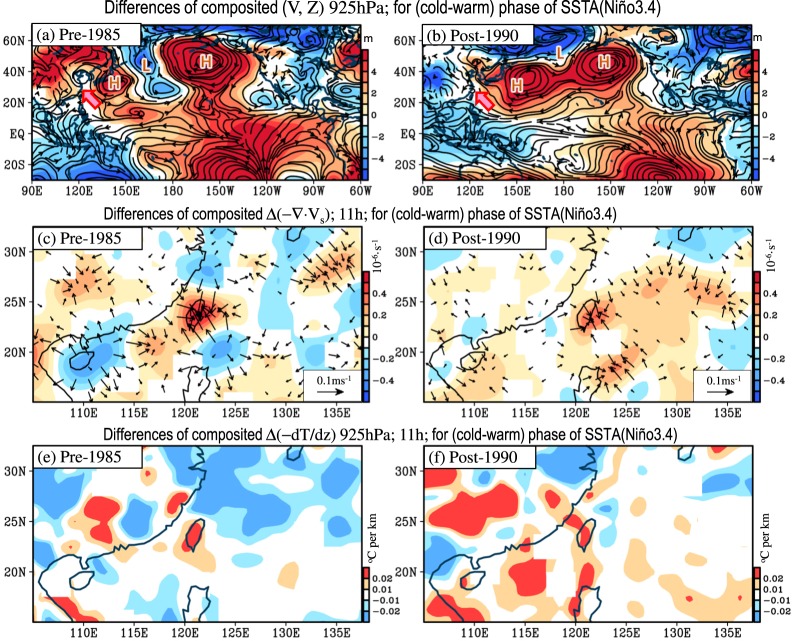
Differences in composited (V, Z) at 925 hPa between the cold (<0.5 STD) and warm (>0.5 STD) years of the filtered SSTA(Niño3.4) (red line in Fig. 3b) during two time periods: (**a**) pre-1985 (i.e., JJA of 1961–1985) and (**b**) post-1990 (i.e., JJA of 1990–2012). In (**a**,**b**), “H” and “L” denote the high (i.e., anticyclonic) and low (i.e., cyclonic) systems described in the manuscript, respectively. The arrow in (**a**,**b**) indicates the southerly wind anomaly. (**c**,**d**) are similar to (**a**,**b**), respectively, but for Δ(−∇·V_s_) at 11 h. (**e**,**f**) are similar to (**c**,**d**), respectively, but for the thermal instability ${\rm{\Delta }}(\,-\,\frac{dT}{dz})$ at 925 hPa, 11 h. Only the areas with values that pass the 90% confidence interval are shown.

It is hypothesized that these atmospheric conditions that are favorable for the formation of CAR in Taiwan have been enhanced (weakened) in the colder (warmer) phase of SSTA(Niño3.4) during JJA of 1961–2012 to facilitate (suppress) CAR formation in Taiwan. Simulating the circulation changes caused by the SSTA changes, Wang *et al*.^14^ showed that the warming in SSTA(Niño3.4) can generate a wave train pattern with a cyclone over the North Pacific east of the international dateline, an anticyclone west of the dateline and a cyclone over the area east of Taiwan (see Fig. 14 of Wang *et al*.^14^). A comparison of the wave train pattern of Wang *et al*.^14^ and the features shown in Fig. 4b shows a similar wave train pattern but with the opposite sign (i.e., an anticyclone over the North Pacific east of the international dateline, a cyclone west of the dateline and an anticyclone over the area east of Taiwan) in Fig. 4b. This implies that the atmospheric circulations in response to the warming (cooling) in SSTA(Niño3.4) might be unfavorable (favorable) for CAR formation in Taiwan. To clarify this hypothesis, we examine the differences in the composited low-level circulations between the colder and warmer phases of SSTA(Niño3.4) (Fig. 5a,b). Notably, to show whether the maintenance mechanism changed after the 1990s, the following examination of Fig. 5 is separated into two time periods: pre-1985 and post-1990. As shown in Fig. 5a,b, a wave train pattern with an anticyclone over the North Pacific east of the international dateline, a cyclone west of the dateline and an anticyclone over the area east of Taiwan is enhanced (weakened) in the colder (warmer) phase of SSTA(Niño3.4); this feature, which is similar to that in Fig. 4b, occurs in both the pre-1985 and post-1990 periods. Such an enhanced (weakened) subtropical high over the area east of Taiwan around (150°E, 25°N) is one of the important factors that facilitates (suppresses) CAR formation in Taiwan during the colder (warmer) phase of SSTA(Niño3.4).

The possible relationship between the enhanced WNPSH and the change of large-scale wind convergence is discussed below. Chen^44^ examined the location of large-scale wind convergence in relation to the Northern Hemisphere summer stationary wave and noted that the large-scale wind convergence (i.e., active upward motion) is generally observed over the EASM region at approximately 120°E coupled with a subtropical high system in the east and a subtropical low system in the west. Based on these findings of Chen^44^, one can infer from Fig. 5a,b that Taiwan is in the area with intensified (suppressed) large-scale wind convergence during the colder (warmer) phase of SSTA(Niño3.4). Such an intensified (suppressed) large-scale wind convergence can provide more (less) large-scale dynamical lifting to facilitate (suppress) CAR formation in Taiwan^5^.

Notably, the enhancement of the WNPSH in Fig. 5b covers a larger area than that in Fig. 5a; this might be because in addition to SSTA(Niño3.4), the interannual variation of the WNPSH is also more strongly regulated by the SSTA over the equatorial central Pacific and the maritime continent after the 1990s^25^. Despite the differences between the magnitudes of the enhancement of the WNPSH shown in Fig. 5a,b, we find that under the modulation of the enhanced (weakened) subtropical high over the area east of Taiwan, the local surface wind convergence (Fig. 5c,d) and local thermal instability (Fig. 5e,f) in Taiwan were all enhanced (weakened) during the colder (warmer) phase of SSTA(Niño3.4) in both the pre-1985 and post-1990 periods. Following Huang *et al*.^5^, we select 11 h as the time step to represent the changes in daytime surface wind convergence and thermal instability in Taiwan. The related examination of the 21-year running correlation between the filtered daytime surface wind convergence in Taiwan and SSTA(Niño3.4) (Supplementary Fig. S5a,b) shows that the negative relationship between them is present in JJA throughout 1961–2012; this is also true for the relationship between the filtered daytime thermal instability in Taiwan and SSTA(Niño3.4) (Supplementary Fig. S5c,d). Altogether, the enhanced (weakened) subtropical high over the area east of Taiwan, the enhanced (weakened) local surface wind convergence and the enhanced (weakened) local thermal instability over Taiwan during the colder (warmer) phase of SSTA(Niño3.4), which are present in both the pre-1985 and post-1990 periods, are suggested to be the factors that are responsible for the consistent negative relationship between the filtered CAR frequency in Taiwan and SSTA(Niño3.4) during JJA of 1961–2012.

It should be mentioned that, except for the remote SSTA(Niño3.4), the local SSTA should also play an important role in modulating the interannual variation of CAR frequency in Taiwan. For example, many studies^4,5^ have demonstrated that the colder SSTA around Taiwan can lead to a larger land-sea thermal contrast with a structure of warmer land and colder ocean during the daytime, leading in turn to a stronger sea-breeze convergence around Taiwan. The enhanced sea-breeze convergence can provide more local dynamical lifting to facilitate the CAR formation in Taiwan (i.e., increase the CAR frequency). This phenomenon explains why, in Fig. 3a, the local SSTA around Taiwan and the CAR frequency in Taiwan are negatively correlated. However, one should be aware that because the CAR formation in Taiwan is very complex and is affected by various factors together (including the orographic effect, the island-scale wind circulation, the large-scale wind circulation, etc.)^11^, it is difficult for the current study to quantitatively judge which SSTA (local or remote) is more important based only on the observational results. More analyses (e.g., model simulations) are suggested in the near future to clarify this issue.

### Mechanism for the relationship between CAR intensity and SSTA(Niño3.4)

**Figure 6:**
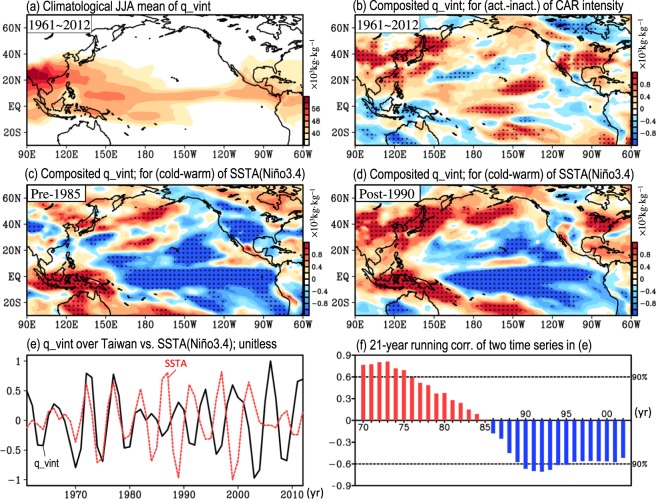
(**a**) Climatological mean of the vertically integrated specific humidity (q_vint) averaged during JJA of 1961–2012. (**b**) Differences in composited q_vint between the active and inactive years of the filtered CAR intensity in Taiwan (black line in Fig. 3d) during JJA of 1961–2012. (**c**,**d**) are similar to (**b**) but for the difference in the composited q_vint between the cold and warm years of the filtered SST(Niño3.4) (red line in Fig. 3d) during two periods: (**a**) pre-1985 and (**b**) post-1990. (**e**) Time series of filtered q_vint over Taiwan (black line) and filtered SSTA(Niño3.4) (red line). (**f**) The 21-year running correlation between the two time-series shown in (**e**). In (**b**–**d**), only the areas with values that pass the 90% confidence interval are dotted. The variables in (**e**) are unitless (i.e., divided by the related maximum amplitude during JJA of 1961–2012).

Next, we focus on explaining causes of the changing relationship between the filtered CAR intensity in Taiwan and SSTA(Niño3.4) in the late 1980s. Previous studies^5–7^ have noted that the more moisture that is supplied to the areas near Taiwan, the more active the CAR intensity in Taiwan is. Consistent with these studies, we note from the comparison between Fig. 6a (i.e., the climatology) and Fig. 6b (i.e., the differences between the active and inactive years of CAR intensity) that the vertically integrated moisture (i.e., q_vint) over the areas near Taiwan is stronger (weaker) during the active (inactive) phase of the CAR intensity in Taiwan. However, when examining the differences in the composited q_vint between the colder and warmer phases of SSTA(Niño3.4), we find that the moisture supply near Taiwan was weakened (enhanced) in the colder (warmer) phase of SSTA(Niño3.4) before the mid-1980s (Fig. 6c), whereas the opposite situation is found after the late 1980s (Fig. 6d). This changing relationship between the interannual variations of moisture supply over the areas near Taiwan and SSTA(Niño3.4) in the mid- to late 1980s is further confirmed by the related examinations of the filtered time series (Fig. 6e) and the 21-year running correlation (Fig. 6f). This change is believed to have led to the changing relationship between the interannual variation of the filtered CAR intensity in Taiwan and SSTA(Niño3.4) in the late 1980s.

The possible linkage between the features revealed in Figs 6 and 5 is discussed below. During the pre-1985 (post-1990) period, the moisture supplies around Taiwan are smaller (larger) in the colder phase than those in the warmer phase of SSTA(Niño3.4); thus, the formation of CAR would be more (less) difficult than usual. In this case, to be able to form the CAR in Taiwan in the colder phase of SSTA(Niño3.4), the magnitude of dynamical lifting forcing (e.g., island-scale wind convergence and thermal instability) would have to be larger during the pre-1985 period than during the post-1990 period. Consistent with this inference, we note in Fig. 5c–f that the sea-breeze convergence and the thermal instability tend to be stronger in Taiwan during the pre-1985 period than during the post-1990 period.

**Figure 7:**
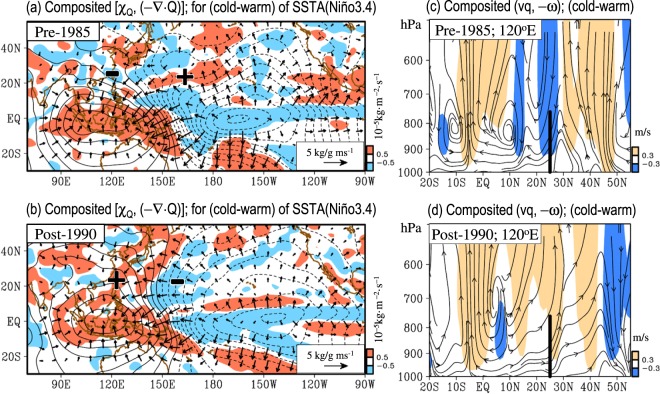
Differences in composited [χ_Q_ (contour), (−∇·Q) (shaded)] between the cold and warm years of SSTA(Niño3.4) during two periods: (**a**) pre-1985 and (**b**) post-1990. In (**a**–**b**), “+” and “−” symbols are added to help indicate the positive and negative values of regional changes, respectively, as discussed in the manuscript. (**c**,**d**) are similar to (**a**,**b**) but for the difference in composited vertical cross-section of the meridional component of the moisture flux (i.e., vq) superimposed on the vertical motion (i.e., −ω; positive values represent upward motion). The moisture transport vectors are added in (**a**,**b**). The location of Taiwan is marked with the black line in (**c**,**d**).

What causes the changing relationship between the interannual variations of moisture supply over the areas near Taiwan and SSTA(Niño3.4)? Notably, Fig. 7a,b show that the relationship between the horizontal moisture flux transport over the “tropical region” and SSTA(Niño3.4) did not change substantially between the pre-1985 and post-1990 periods. In both periods, more (less) water vapor was transported from the Niño3.4 region to the western tropical Pacific region during the colder (warmer) phase of SSTA(Niño3.4) (see Fig. 7a,b). However, in the areas near Taiwan (i.e., the subtropical region of East Asia; indicated by the black solid line in Fig. 7c), one can observe that in relation to the enhanced (weakened) convergence of water vapor flux over the western tropical Pacific, an enhanced (weakened) divergence of water vapor flux and downward (upward) motion occurred over Taiwan during the colder (warmer) phase of SSTA(Niño3.4) during the pre-1985 period (see Fig. 7a,c). In contrast, during the post-1990 period, the moisture flux convergence (divergence) expanded northward to cover most of (10°S-40°N, 120°–130°E), including Taiwan, during the colder (warmer) phase of SSTA(Niño3.4) (Fig. 7b). As a result, the upward motion is more (less) active over Taiwan in the colder (warmer) phase of SSTA(Niño3.4) (Fig. 7d).

It should be mentioned that the enhanced (weakened) moisture flux convergence over the areas near Taiwan during the colder (warmer) phase of SSTA(Niño3.4) after 1993 was also noted by Chen *et al*.^6^, which supports the finding shown in Fig. 7b,d. We propose that the observed changing relationship between the filtered moisture flux convergence around Taiwan and SSTA(Niño3.4) in the late 1980s might be attributed to the changes in the relationship between subtropical SSTA over the East-Asia-Western-North-Pacific (EAWNP) region and SSTA(Niño3.4). The explanation is given below.

By comparing Figs 7a,b and S6, we note that the area with positive (negative) value of moisture flux convergence is coherent with the area that has relative warmer (colder) SSTA than the nearby ocean. During the pre-1985 period, the SSTA over the area at approximately 20°N, 160°E is warmer than the SSTA around Taiwan in the colder phase of SSTA(Niño3.4) (see Fig. S6a). Consistent with these subtropical SSTA patterns, a moisture flux convergence (marked by “+”) is located at approximately 20°N, 160°E, and a moisture flux divergence (marked by “−”) is located around Taiwan (see Fig. 7a). In contrast, during the post-1990 period, a relative colder SSTA coupled with a moisture flux divergence is observed at approximately 20°N, 160°E, and a relative warmer SSTA coupled with a moisture flux convergence is revealed around Taiwan in the colder phase of SSTA(Niño3.4) (see Figs S6b and 7b). Obviously, the distributions of regional SSTA do modulate the distributions of regional moisture flux convergence/divergence. Furthermore, because the regional SSTA pattern in relation to the variations of SSTA(Niño3.4) has changed between the pre-1985 and post-1990 periods, the regional moisture flux convergence/divergence pattern around Taiwan has also changed the relationship with SSTA(Niño3.4).

Regarding why the regional SSTA has a different relationship with SSTA(Niño3.4) between the pre-1985 and post-1990 periods, we believe this difference might be related to the shape change of SSTA patterns. As seen in Fig. S6, the warm subtropical SSTA pattern in relation to the colder phase of SSTA(Niño3.4) is distributed in a more tilted direction during the pre-1985 period (i.e., northeast-southwest, as indicated by the dashed arrow in Fig. S6a) but a less tilted direction during the post-1990 period (i.e., north-south, as indicated by the dashed arrow in Fig. S6b). Further investigations (e.g., model simulations) are suggested in the near future to clarify the causes of the shape change of SSTA patterns seen in Fig. S6.

## Conclusion and Discussion

In this study, the relationship between the 4-to-8-year interannual variations of CAR activity (including frequency and intensity) in Taiwan and SSTA(Niño3.4) during JJA of 1961–2012 are identified and explained. This 4-to-8-year interannual variation signal was first indicated by Huang *et al*.^5^, but the details of the relationship of the signal variations with SSTA(Niño3.4) and underlying physical mechanisms have been documented for the first time by the current study. Our analyses show that a consistent negative relationship existed between the interannual variation of CAR frequency in Taiwan and SSTA(Niño3.4) throughout the entire period. In contrast, the relationship between the interannual variation of CAR intensity in Taiwan and SSTA(Niño3.4) changed from positively correlated to negatively correlated in the late 1980s.

**Figure 8:**
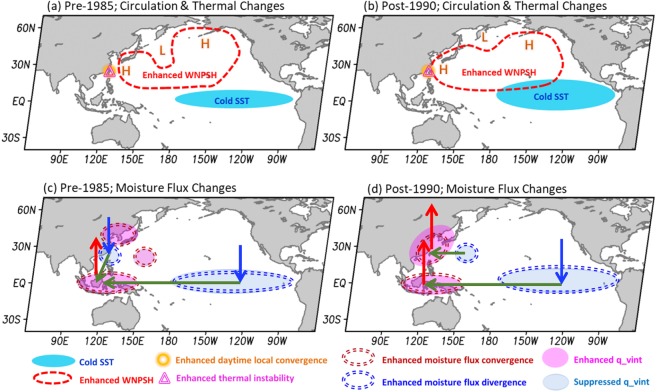
Schematic diagrams of the maintenance mechanisms of the interannual variation of CAR frequency in Taiwan during two periods: (**a**) pre-1985 and (**b**) post-1990. (**c**,**d**) correspond to (**a**,**b**) but for the interannual variation of CAR intensity in Taiwan. In (**c**,**d**), the green, red and blue arrows denote the directions of the horizontal moisture transport, upward motion and downward motion, respectively. The meanings of the other symbols are given below (**c**,**d**).

Figure 8 shows a schematic diagram that summarizes the proposed physical explanations for the relationship between the interannual variations of CAR activity in Taiwan and SSTA(Niño3.4). As shown in Fig. 8a,b, in both the pre-1985 and post-1990 periods, all of the important atmospheric conditions that are favorable for CAR formation in Taiwan (including the enhanced subtropical high over the area east of Taiwan, the enhanced local daytime sea breeze convergence and enhanced daytime thermal instability) are found to be stronger during the colder phase of SSTA(Niño3.4). As a result, a negative relationship between the interannual variation of CAR frequency in Taiwan and SSTA(Niño3.4) occurred in JJA throughout 1961–2012. In contrast, as shown in Fig. 8c (Fig. 8d), our analyses show that the moisture supply over the areas near Taiwan was suppressed (enhanced) during the colder phase of SSTA(Niño3.4) in the pre-1985 (post-1990) period. In response to these changes in the moisture supply, the relationship between the interannual variation of CAR intensity in Taiwan and the SSTA(Niño3.4) changed from positively correlated to negatively correlated in the late 1980s.

Notably, this study is the first to point out the changing relationship between the interannual variation of CAR intensity in Taiwan and SSTA(Niño3.4) in the late 1980s. In addition, we extend the analysis of Chen *et al*.^6^, who focus on the changes in summer thunderstorm activity in Taipei (northern Taiwan) after 1993, to demonstrate that the negative relationship between CAR frequency in Taiwan and SSTA(Niño3.4) existed in JJA over the entire time period of 1961–2012. These findings provide useful information for understanding how the rainfall characteristics in East Asia have changed in response to the changes in SSTA(Niño3.4) over the past several decades. Moreover, because the seasonal forecasts of SSTA(Niño3.4) are regularly issued by several Climate Prediction Centers around the world (http://www.clivar.org/clivar-panels/pacific/enso), the relationships between the characteristics of CAR in Taiwan and SSTA(Niño3.4) that are documented in this study might have some practical implications in terms of seasonal prediction and beyond. This topic deserves further analysis for verification.

## Supplementary information

Relationship between the Interannual Variations of Summer Convective Afternoon Rainfall Activity in Taiwan and SSTA(Niño3.4) during 1961-2012: Characteristics and Mechanisms
