# Supplementary material for: Relationship between the Interannual Variations of Summer Convective Afternoon Rainfall Activity in Taiwan and SSTA(Niño3.4) during 1961–2012: Characteristics and Mechanisms
Source: Sci Rep. 2019 Jun 28;9:9378. doi: 10.1038/s41598-019-45901-w (PMC6598986; doi:10.1038/s41598-019-45901-w)
Supplement: Supplementary file 1 — Relationship between the Interannual Variations of Summer Convective Afternoon Rainfall Activity in Taiwan and SSTA(Niño3.4) during 1961-2012: Characteristics and Mechanisms [file 41598_2019_45901_MOESM1_ESM.pdf]

Supplementary Information for:

Relationship between the Interannual Variations of Summer Convective Afternoon Rainfall Activity in Taiwan and SSTA(Niño3.4) during 1961-2012: Characteristics and Mechanisms

Authors: Wan-Ru Huang*, Ya-Hui Chang and Po-Han Huang

Affiliation: Department of Earth Sciences, National Taiwan Normal University, Taipei, Taiwan R.O.C.

*Corresponding author E-mail: wrhuang@ntnu.edu.tw

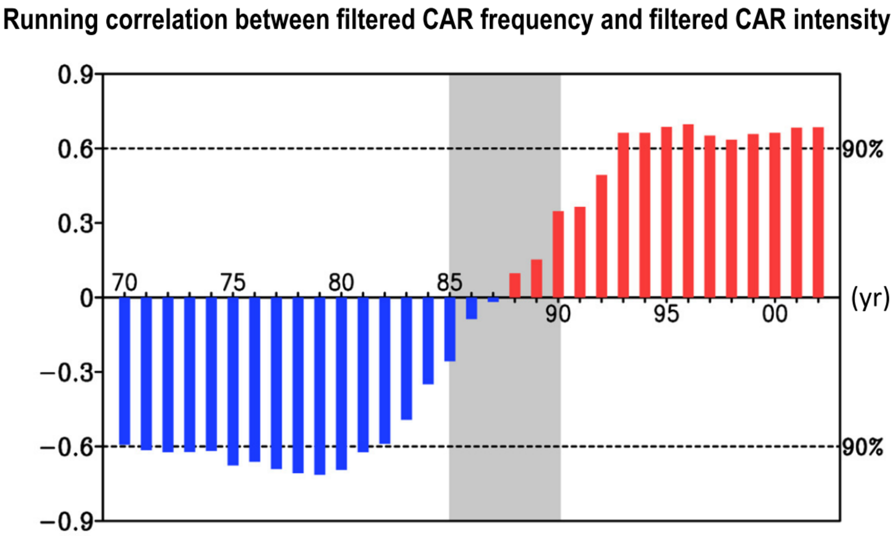

Fig. S1 The 21-year running correlation between the time-series of 4-to-8-year bandpass filtered CAR frequency and CAR intensity shown in Fig. 2. On the x-axis, the year represents the mid-point of the 21-year window (e.g., 70 represents the running window of 1961-1981). The transition zone (between 1985 to 1990; marked in gray) is defined as the 21-year running correlation within the values of ±0.3. The dotted lines represent the values significant at the 90% confidence interval.

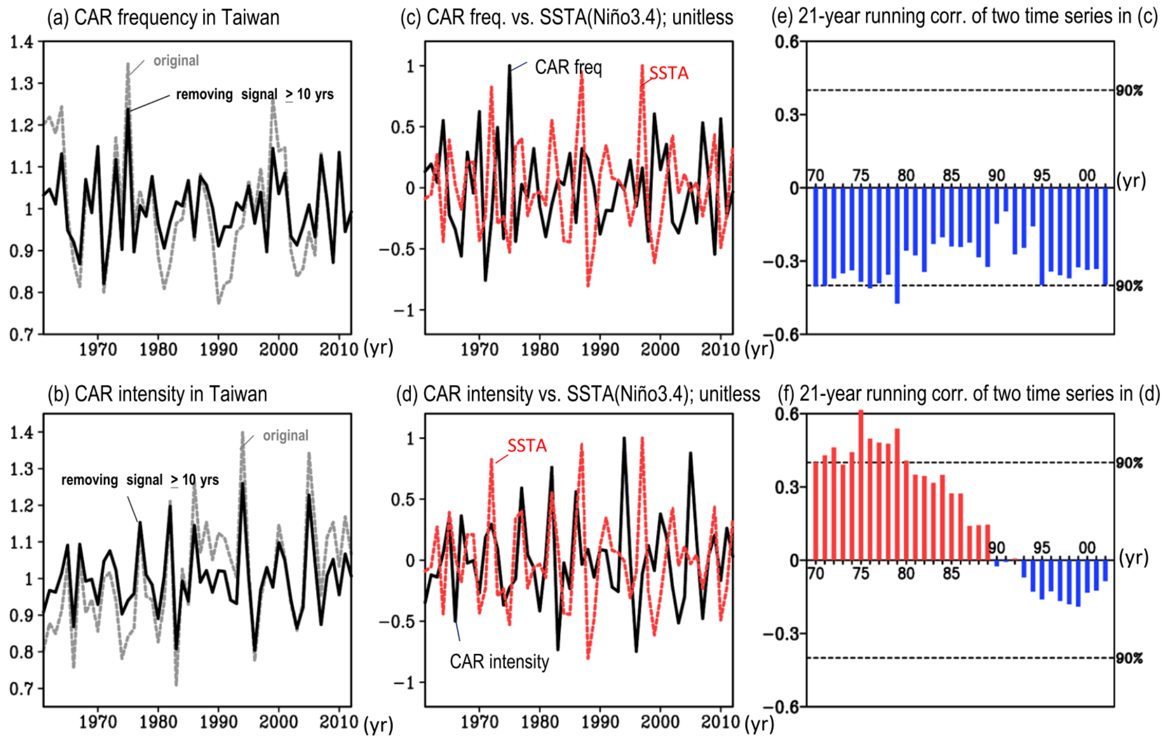

Fig. S2 The total time series (gray lines) and new time series (black lines; i.e., obtained by removing the signals ≥ 10 years from the total time series) of (a) CAR frequency and (b) CAR intensity. (c)-(f) are similar to Figs. 3(b)-(e) in the manuscript except for the illustration of the phase relationships using the time series obtained by simply removing signals ≥ 10 years from the total time series.

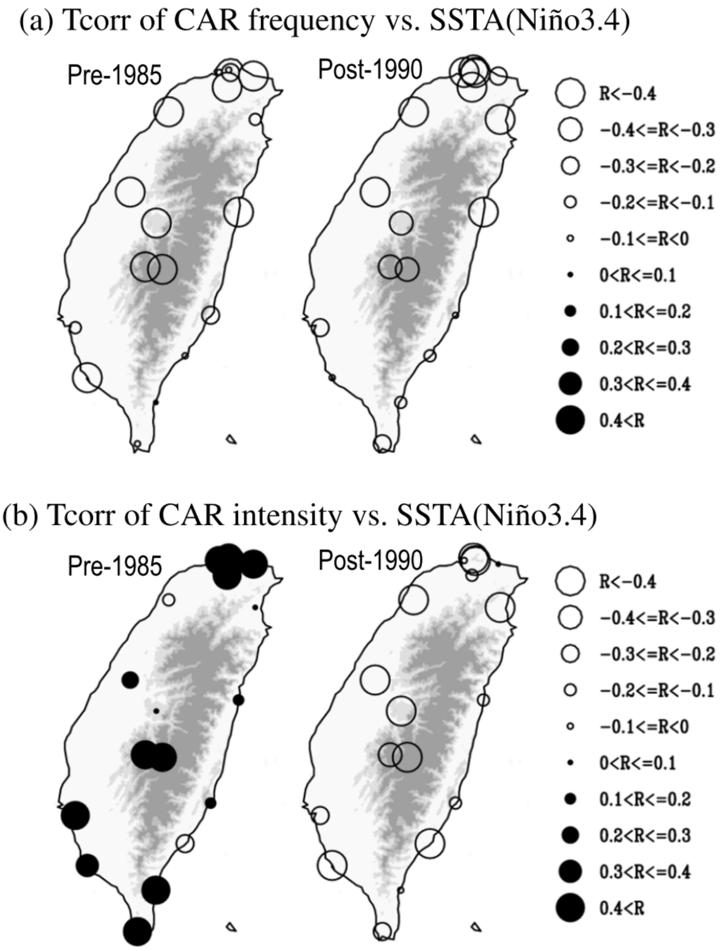

Fig. S3 (a) Temporal correlation (Tcorr) between the 4-to-8-year bandpass filtered CAR frequency for each individual station and SSTA(Niño3.4) during the pre-1985 period (i.e., JJA of 1961-1985; left) and the post-1990 period (i.e., JJA of 1990-2012; right). (b) is similar to (a) except for the Tcorr between the filtered CAR intensity in each individual station and SSTA(Niño3.4). Information and the scale for the circles are given on the right.

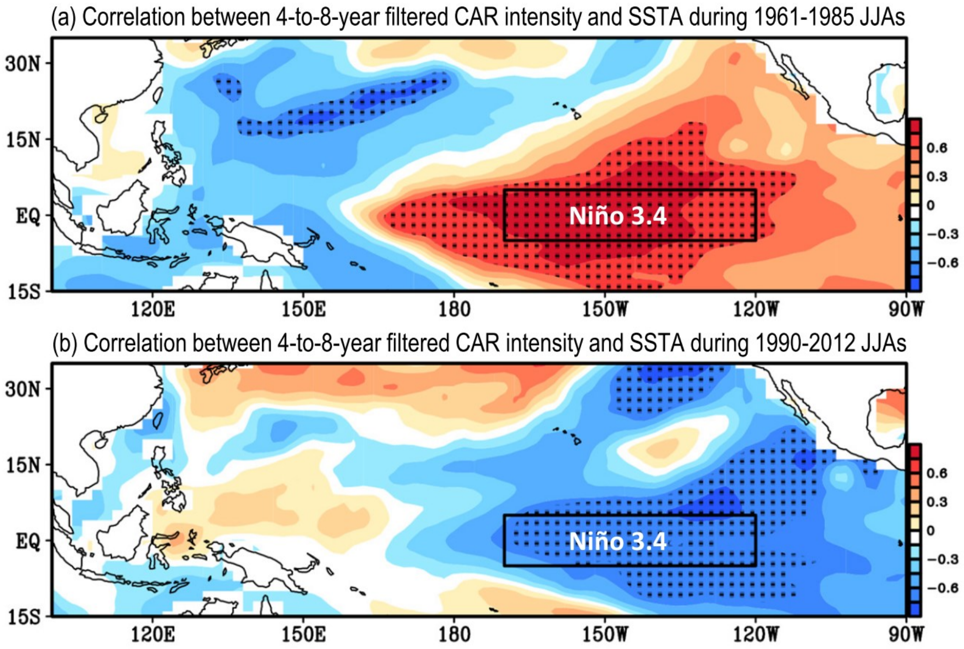

Fig. S4 (a) Horizontal distribution of the correlations between the 4-to-8-year bandpass filtered CAR intensity in Taiwan and SSTA during the pre-1985 period (i.e., JJA of 1961-1985). (b) is similar to (a) but for the correlations during the post-1990 period (i.e., JJA of 1990-2012). In (a)-(b), the areas with correlations that pass the 90% confidence interval are dotted.

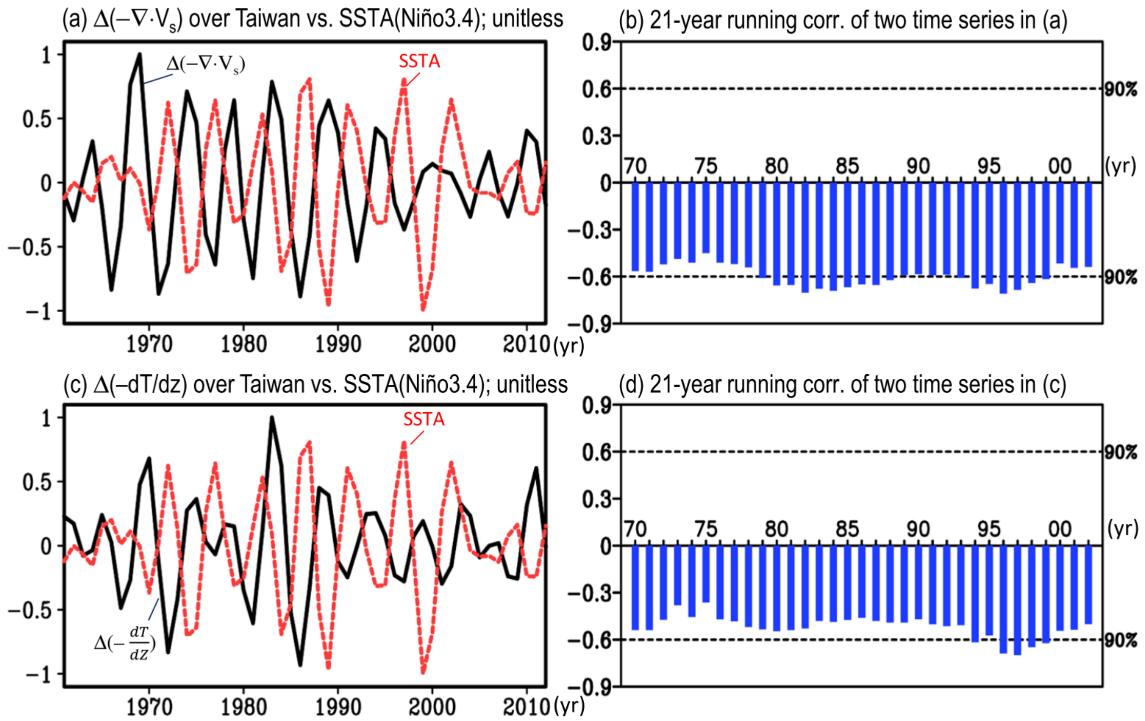

Fig. S5 (a) Time series of filtered $\Delta(-\nabla\cdot V_s)$ at 11 h area-averaged over the domain of Taiwan (120-122.5°E; 22-25.5°N) (black line) and filtered SSTA(Niño3.4) (red line) in JJA of 1961-2012. (b) The 21-year running correlation between the two time-series shown in (a). (c)-(d) are similar to (a)-(b) but for the comparison between the filtered Δ(–dT/dz) at 925 hPa, 11 h, over Taiwan and the filtered SSTA(Niño3.4). The variables in (a)-(c) are unitless (i.e., divided by the related maximum amplitude in JJA of 1961-2012).

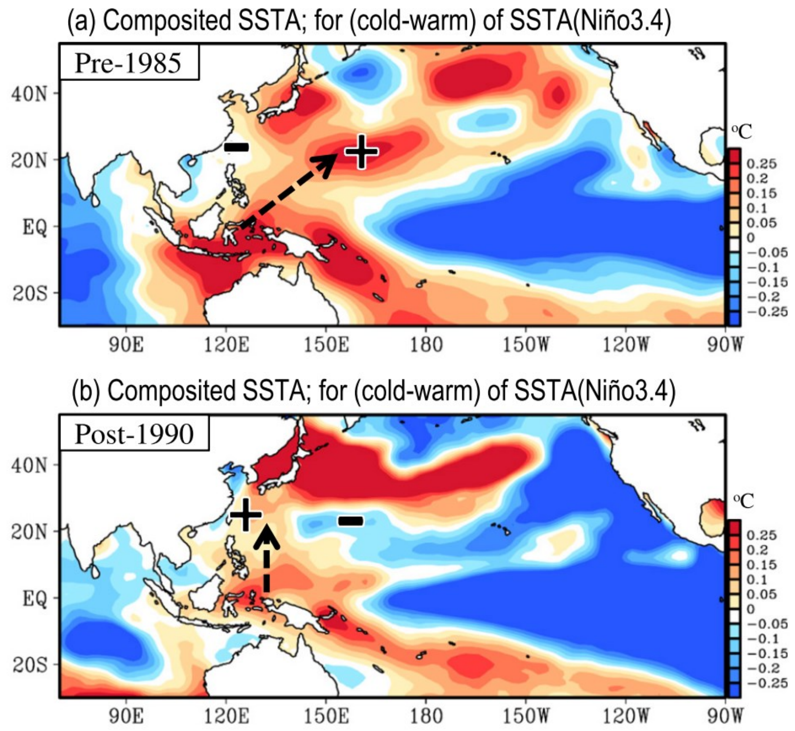

Fig. S6 Differences in composited SSTA between the cold and warm years of SSTA(Niño3.4) during two periods: (a) pre-1985 and (b) post-1990. "+" and "–" symbols are added to indicate the positive and negative values, respectively, of subtropical regional SSTA discussed in the manuscript. The dashed arrows are added to indicate the shape of the distribution of the warm SSTA pattern over the western tropical and subtropical Pacific regions.
